# Multilevel Bayesian analysis of monk parakeet contact calls shows dialects between European cities

**DOI:** 10.1093/beheco/arad093

**Published:** 2023-11-23

**Authors:** Simeon Q Smeele, Stephen A Tyndel, Lucy M Aplin, Mary Brooke McElreath

**Affiliations:** Cognitive & Cultural Ecology Research Group, Max Planck Institute of Animal Behavior, Am Obstberg 1, 78315 Radolfzell, Germany; Department of Biology, University of Konstanz, Universitätsstraße 10, 78464 Konstanz, Germany; Department of Human Behavior, Ecology and Culture, Max Planck Institute for Evolutionary Anthropology, Deutscher Pl. 6, 04103 Leipzig, Germany; Ecoscience, Aarhus University, Nordre Ringgade 1, 8000 Aarhus C, Denmark; Cognitive & Cultural Ecology Research Group, Max Planck Institute of Animal Behavior, Am Obstberg 1, 78315 Radolfzell, Germany; Department of Biology, University of Konstanz, Universitätsstraße 10, 78464 Konstanz, Germany; Cognitive & Cultural Ecology Research Group, Max Planck Institute of Animal Behavior, Am Obstberg 1, 78315 Radolfzell, Germany; Division of Ecology and Evolution, Research School of Biology, The Australian National University, 134 Linnaeus Way, Acton ACT 2601, Australia; Department of Evolutionary Biology and Environmental Science, University of Zurich, Winterthurerstrasse 190, 8057 Zürich, Switzerland; Cognitive & Cultural Ecology Research Group, Max Planck Institute of Animal Behavior, Am Obstberg 1, 78315 Radolfzell, Germany; Department of Human Behavior, Ecology and Culture, Max Planck Institute for Evolutionary Anthropology, Deutscher Pl. 6, 04103 Leipzig, Germany

**Keywords:** Bayesian statistics, communication, culture, dialects, monk parakeet, open-ended vocal learning

## Abstract

Geographic differences in vocalizations provide strong evidence for animal culture, with patterns likely arising from generations of social learning and transmission. Most studies on the evolution of avian vocal variation have predominantly focused on fixed repertoire, territorial song in passerine birds. The study of vocal communication in open-ended learners and in contexts where vocalizations serve other functions is therefore necessary for a more comprehensive understanding of vocal dialect evolution. Parrots are open-ended vocal production learners that use vocalizations for social contact and coordination. Geographic variation in parrot vocalizations typically take the form of either distinct regional variations known as dialects or graded variation based on geographic distance known as clinal variation. In this study, we recorded monk parakeets (*Myiopsitta monachus*) across multiple spatial scales (i.e., parks and cities) in their European invasive range. We then compared calls using a multilevel Bayesian model and sensitivity analysis, with this novel approach allowing us to explicitly compare vocalizations at multiple spatial scales. We found support for founder effects and/or cultural drift at the city level, consistent with passive cultural processes leading to large-scale dialect differences. We did not find a strong signal for dialect or clinal differences between parks within cities, suggesting that birds did not actively converge on a group level signal, as expected under the group membership hypothesis. We demonstrate the robustness of our findings and offer an explanation that unifies the results of prior monk parakeet vocalization studies.

## Teaser

Dialects, that is, regional variation in vocalizations, in animals provide one of the most widespread examples of animal culture. We studied dialects in a vocally flexible parrot, the monk parakeet, in order to understand the mechanisms of how they form. For example, it is often thought that dialects form as a mechanism to identify group members. Instead, we found evidence that dialects most likely occur because of random cultural processes.

## INTRODUCTION

Differences in vocalizations between groups or populations have been identified within multiple animal species. Such geographic variation in vocalizations has provided some of the strongest evidence for vocal learning and animal culture (Marler and Tamura 1962; Catchpole and Slater 2003; Podos and Warren 2007; Aplin 2019). In particular, patterns of vocal variation in songbirds have been the focus of decades of intensive research (Slater 2003). In songbirds, song is primarily used to defend territories and attract mates (Krebs and Kroodsma 1980; Kroodsma and Byers 1991; Catchpole and Slater 2003), and is often exclusively learned early in development. Coupled with vocal convergence and conformity (Lachlan et al. 2018), this early flexibility can result in highly stable and localized dialects. For example, male new world sparrows (*Passerllidae*) produce complex songs that form clear geographic dialects (Williams et al. 2013; Lachlan et al. 2018). These dialects are maintained over long periods of time and may play an important function in species recognition and mate choice (Slater 2003; Lachlan et al. 2018). Furthermore, the way dialects are structured can depend heavily on behavior and social structure. This is supported by examples of species that have limited migration and dispersal between populations, which show a gradual change in vocal differentiation across a geographical clinal gradient (Irwin et al. 2008). However, the study of vocal variation in open-ended vocal production learners outside the context of bird-song is relatively understudied and the mechanisms leading to emergent dialect or clinal patterns in these cases are poorly understood (Wright and Dahlin 2018).

Open-ended vocal production learning refers to the ability to modify or change produced vocalizations throughout adulthood (Janik and Slater 1997; Beecher and Brenowitz 2005; Janik and Knörnschild 2021). Open-ended vocal production learning has evolved in several taxonomic groups including bats, cetaceans, and three main groups of birds: hummingbirds (*Trochilidae*), passerines (i.e., *Corvidae*, *Fringillidae*, *Sturnidae*), and parrots (*Psittaformes*). Many parrot species show geographic variation in their contact calls (Wright 1996; Wright and Dahlin 2018), and, in captive studies, are able to actively converge their vocalizations across long (multiple weeks) time scales (Hile et al. 2000). This observation of group convergence has been hypothesized to lead to group-level vocal signatures (Dahlin et al. 2014). In addition to long time scales, parrots can also rapidly modify their calls (i.e., within seconds) (Vehrencamp et al. 2003; Balsby and Bradbury 2009; Scarl and Bradbury 2009; Thomsen et al. 2019) depending on specific social context (i.e., addressing flock members (Balsby et al. 2012)). This extreme rapid flexibility could be another possible mechanism leading to overarching geographic variation (Barker et al. 2021).

Several hypotheses have been proposed to explain patterns of geographic vocal variation in open-ended vocal production learners such as parrots. The *group membership hypothesis* posits that vocal dialects serve a functional purpose of increased recognition of group members and possibly foraging efficiency within social groups (Payne 1981; Bradbury et al. 1998; Podos and Warren 2007; Sewall et al. 2016). In support of this hypothesis, a wide range of studies have found that some parrot species (Wright and Dorin 2001; Vehrencamp et al. 2003; Dahlin et al. 2014), bats (Knörnschild et al. 2012), and dolphins (Janik and Slater 1998) appear to use calls to strengthen social bonds in groups. Under this framework, particular call types, and/or dialects could undergo social selection, allowing for stable call types (Wright 1996). In terms of observable predictions, we would propose that this active process of group convergence should manifest as group signatures at small geographic scales, with this scale further depending on group size and social structure. Along the same lines, if populations demonstrate large degrees of fission–fusion dynamics, this could possibly lead to a clinal gradient, where vocalizations produced in close geographic proximity sound more similar than those produced further apart (Bradbury et al. 2001). This relies on the assumption that animals would have limited dispersal, and would be more likely to spend time in areas in close geographic proximity, versus those further away.

The *cultural drift hypothesis* proposes that vocal variation is the result of passive cultural processes, with either copying errors or innovations combined with neutral or directional cultural evolution that allows for groups to diverge (Payne 1978; Williams et al. 2013; Williams and Lachlan 2022). Previous research suggests that sexual (Nowicki, Peters, and Podos 1998) and social selection (Lachlan et al. 2018) both represent likely selective pressures in songbird species. In open-ended learning species such as parrots, contact calls are likely not subject to sexual selection (Bradbury and Balsby 2016). Isolation and cultural drift combined with social selection, therefore appears to be the most plausible explanation for the patterns of vocal variation observed in many species. For example, crimson rosella (*Platycercus elegans*) (Ribot et al. 2012) and St. Lucia parrots (*Amazona versicolor*) (Martínez and Logue 2020) both demonstrate dialect boundaries that correspond to barriers to movement. Unlike the *group membership hypothesis*, *the cultural drift hypothesis* does not necessarily require selection for convergence at the group level. Instead, we would expect to observe dialects across isolated geographic regions, likely at larger scales where boundaries exist that isolate populations.

In contrast to the *group membership hypothesis*, the *individual signature hypothesis* posits that individuals actively modify their vocalizations to try and sound as distinct from one another as possible (Nowicki and Searcy 2014). In this scenario, we would not necessarily expect to observe geographic vocal variation, despite the social learning of vocalizations. This is because the drive for individual distinctiveness may lead to increased variation within groups (Gillam and Chaverri 2012), making any effect of cultural drift between populations difficult to detect. This type of pattern has been observed in other open-ended learning species such as dolphins (Oswald et al. 2021), and parrot species such as green rumped parrotlets (*Forpus passerinus*) (Berg et al. 2011) and monk parakeets (Smith-Vidaurre et al. 2020). However, the *individual signature hypothesis* is not necessarily mutually exclusive with the *group membership hypothesis*. Indeed there is evidence that some species can maintain individual signatures while also maintaining strong group level signatures (Wright 1996, 1996; Thomsen et al. 2013). The precise mechanism that causes individual signatures to outweigh dialects versus having strong individual signatures in concert with strong dialect boundaries remains unclear.

Monk parakeets (*Myiopsitta monachus*) are an excellent study system to elucidate the processes that lead to geographic vocal variation in open-ended vocal learners. Monk parakeets have a large invasive range across Europe and North America (Forshaw and Cooper 1989), where populations are largely concentrated in cities, often with little movement between them (Edelaar et al. 2015; Postigo et al. 2019). Importantly, several features of monk parakeet population substructure allow for close study of geographic vocal variation patterns at multiple scales. They nest in single or compound nests, the latter containing multiple nests, each with one or multiple chambers per pair (SQS personal observation). Nest openings correspond to nest chambers, which can serve as a proxy for population size. These nest structures occur in larger nesting colonies. The term colony is often defined as one or more nest structures located within 200 m of each other (see Reed et al. (2014), Supplementary Materials). In cities and invasive populations, these nesting colonies are often located within parks or other green areas, clearly delineated from other colonies, although with potential between-park movement and dispersal (Borray-Escalante et al. 2023; Bucher et al. 1990). A recent study in the native range of monk parakeets found evidence that individual signatures outweighed any emergent dialects (Smith-Vidaurre et al. 2020). Interestingly, regional dialects between cities have been observed in the invasive populations of monk parakeets in the United States (Buhrman-Deever et al. 2007).

In the current study, we aim to assess these competing hypotheses by examining patterns of vocalizations across parks and cities in the invasive range of monk parakeets in Europe. Because most European populations of monk parakeets have comparable genetic compositions (Edelaar et al. 2015), it allows us to consider the influence of cultural processes rather than potential genetic differences between the populations. Our populations contain many sub-populations (i.e., parks) making it possible to conduct a two-level comparison with many replicates. If dialects or clinal variation are found at the park level, selection for call sharing with other group members is likely at play, lending credence to vocal convergence via the g*roup membership hypothesis*. Of course, if movement between parks is low, one could not rule out the possibility of founder effects and/or cultural drift. If dialects exist only at the city level, it would suggest a cultural founder effect and/or cultural drift, similar to that often observed in songbirds (Baker and Jenkins 1987; Lachlan et al. 2018).

## METHODS

### Study system

Monk parakeets are a medium-sized colonially nesting parrot. While native to South America, they have been transported by the pet trade across the world and have established large invasive populations in several European countries including Spain, Belgium, Italy, and Greece. These populations are usually clustered in cities and towns, often with relatively little dispersal between them (Dawson Pell et al. 2021). Monk parakeets in Europe breed from March to August and roost in their nests year-round (Senar et al. 2019). Nests are often highly spatially clustered, with several nest chambers per nest, several nests per tree and trees often clustered together (Eberhard 1998). Population sizes vary within and between cities and parks, with estimates ranging between one nest chamber in Thisio park, Athens to 99 in Gendarmerie School Park, Athens.

### Data collection

**Figure 1:**
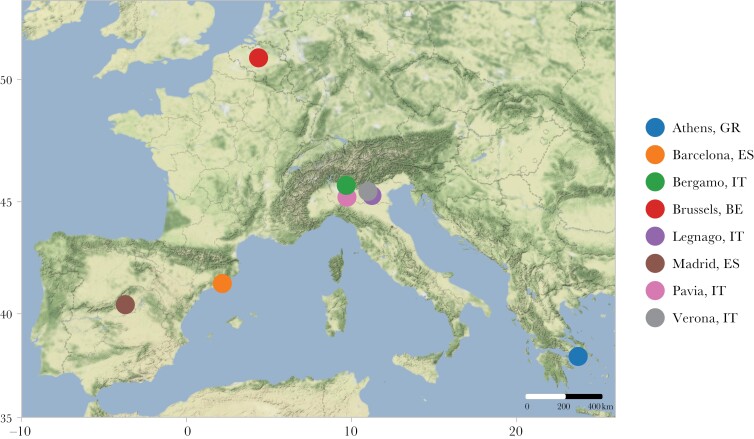
Map of study locations. Map created using ggmap (Kahle and Wickham 2013), ggrepel (Slowikowski 2021), and ggsn (Santos Baquero 2019). Map tiles by Stamen Design, under CC BY 3.0. Data by OpenStreetMap, under ODbL.

We collected vocalizations from monk parakeets in 39 parks across eight cities in four countries: Athens, Barcelona, Bergamo, Brussels, Legnago, Madrid, Pavia, and Verona in November 2019 (see Table 1 for sampling effort per park, see Figure 1 for sampling area, and see Supplementary Materials for maps of parks within cities). Vocalizations were opportunistically recorded between sunrise and sunset with a Sennheiser K6 + ME67 microphone and either a Sony PCM M10 or Sony PCM D100 recorder. Recordings were made at a distance between 1 and 20 m and lasted 20 min or until the bird moved away. If calls could be assigned with certainty to a focal bird this was verbally annotated.

**Table 1:** Recording location surveyed in this study. Number of days represents how many days the parks were visited. Not all recording sessions were entire days. Number of calls represents how many calls were included in the final analysis.

City	Park	Number of days	Number of calls	Number of nest openings
Athens	Oluf Palme Playground	3	35	9
Athens	National Garden	4	287	49
Athens	Alsos Ilision	2	52	10
Athens	Gendarmerie School Park	3	86	99
Athens	Thissio Park	1	2	1
Barcelona	Parc de la Ciutadella	3	85	33
Barcelona	Jardins del Turo del Putxet	1	98	1
Barcelona	Jardins de Ghandi	1	2	4
Barcelona	Jardins de Josep Trueta	1	20	7
Barcelona	Parc Grande de Sant Martéí?	1	44	54
Barcelona	Jardins de la Maternitat	1	19	11
Bergamo	Faunistic Park Le Cornelle	2	456	26
Brussels	Parc de Forest	4	559	96
Brussels	Ten Reuken	2	19	NA
Brussels	Avenue Louise	1	6	7
Brussels	Tenenbosch Park	1	10	1
Brussels	Place Guy D'Arezzo	2	107	13
Legnago	Legnago	2	345	10
Madrid	Parque de el Ritero	1	13	NA
Madrid	Parque de Berlin	3	218	65
Madrid	Lago Casa del Campo	2	91	18
Madrid	Parque Azorin	2	141	55
Madrid	Parque Emperatriz Maria de Austria	1	45	NA
Madrid	Parque Infantil Portalegre	1	9	6
Madrid	Quintos de Molinos	1	10	2
Madrid	Parque Alfredo Kraus	1	5	10
Pavia	Oasi di Sant'Alessio	1	756	34
Verona	Parco Natura Viva	1	110	37

Although individuals were not identifiable across recordings, whenever possible we recorded the vocalizing individual with a unique ID within a recording. We also included recordings when the vocalizing individual was not assigned a unique ID. In order to avoid assigning a unique ID to each vocalization made by an unidentified individual, we grouped them by 5 min intervals of recording, assuming recordings during that time span came from one individual. Some recordings were also videotaped with a Philips HC-V777EG-K to allow assignment of calls during processing. We tested how this incorrect pooling might have affected the results in a sensitivity analysis (see Supplementary Materials).

### Data processing

Raw recordings were first imported to Raven Lite 2.0 (Cornell Lab of Ornithology, NY 2016). We manually selected the start and end times of all vocalizations with reasonable signal to noise ratio and annotated the caller ID and behavior if available. Using a custom script in R (R Core Team 2021), all selected calls from Raven Lite 2.0 were clipped and high quality spectrograms were created (see Data availability statement). All spectrograms were then manually inspected and calls that were considered to be poor quality were removed.

**Figure 2:**
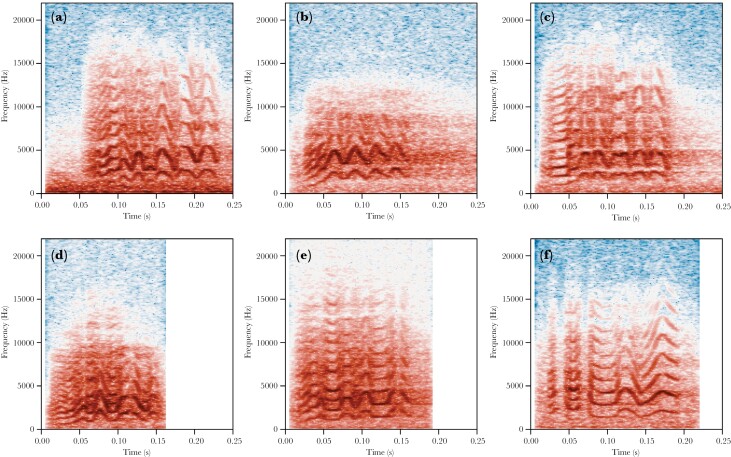
Examples of four contact call variants. a) typical, b) four triangle, c) ladder start, d) ladder middle, e) ladder multiple, and f) mix alarm.

The remaining calls were categorized as either contact calls (tonal calls with at least three peaks in their frequency modulation) or other calls. Contact calls were further manually sorted into six variants: *typical* (call with at least four frequency modulated components), *four triangle* (stereotyped call with four triangular shaped frequency modulated components), *ladder start* (call with low frequency harmonic in the first component), *ladder middle* (call with low frequency harmonic in the middle of the call), *ladder multiple* (call with multiple low frequency harmonic components), and *mix alarm* (call with frequency modulated components mixed with amplitude modulated components). For examples of these variants, see Figure 2. We chose to use a structural definition to designate call types rather than a behavioral one, since most recordings for which behavioral information was available were of single perched individuals (Smith-Vidaurre et al. 2020).

To assess whether our categorizations of call variants were reproducible, we created a randomized sample of 1000 calls from our dataset, including both contact and non-contact calls. We then asked an independent observer to classify the calls, and we assessed both how the observer’s classifications of contact calls versus non-contact calls, and how the observer’s classifications of contact call variants compared to our own. The agreement between our own observations of contact versus non-contact calls and the independent observers’ observations was very strong (Kappa statistic, k = 0.83, Z = 26.2, %-agree = 91.6). The agreement between our classifications of contact call variants and the independent observers’ classifications was moderately strong (Kappa statistic, k = 0.59, Z = 35.6, %-agree = 74.3).

All good quality contact calls were saved as separate sound files and imported to Luscinia v2.16.10.29.01 (Lachlan 2007). Using Luscinia’s algorithm, we traced the fundamental frequency semi-manually. Some calls could not be traced well and were excluded (28%). The fundamental frequency traces were imported to R and smoothed in two steps to get rid of small errors. First, gaps where Luscinia could not detect the fundamental frequency were filled with a straight line from the last detected point to the first detected point after the gap. Then smooth.spline (*stats*) was used with spar = 0.4 to remove outliers. Traces were visually inspected to ensure proper fit.

We used dynamic time warping (DTW) to measure similarity between all pairs of contact calls. This algorithm takes two time series and measures the optimal similarity between them (Bellman and Kalaba 1959). We used the function *dtw* from the package *dtw* (Giorgino 2009) to run DTW on the fundamental frequency traces. We normalized and log transformed the resulting distance matrix. To represent each call as a single point in two-dimensional space, we ran a principal coordinate analysis (PCO) using the function *pcoa* from the package *ape* (Paradis and Schliep 2019). To verify the robustness of our DTW-PCO analysis, we also obtained a distance matrix using spectrographic cross correlation using the entire spectrogram. We also used uniform manifold approximation and principal component analysis for dimension reduction (see Supplementary Materials). All approaches gave similar results.

### Statistical analysis

We used a Bayesian multilevel model to test how much variation in PC1 and PC2 was explained by the two geographic levels of interest, park and city. Both were included as varying effects. To control for pseudoreplication, we included the verbally annotated IDs whenever possible as varying effects as well. When IDs were not available, we grouped all calls occurring in the same 5 min interval as one individual. We conducted a sensitivity analysis to test how well this approach could mitigate the effects of pseudoreplication (see Supplementary Materials). The full model structure for PC1 (standardized) is as follows:


$$PC1∼normal\left(\mu_{obs},\sigma_{obs}\right)$$

$$\mu_{obs\left[i\right]}=\alpha_{city\left[i\right]}+\alpha_{park\left[i\right]}+\alpha_{ind\left[i\right]}$$

$$\alpha_{city}∼normal\left(\mu_{city},\sigma_{city}\right)$$

$$\alpha_{park}∼normal\left(0,\sigma_{park}\right)$$

$$\alpha_{ind}∼normal\left(0,\sigma_{ind}\right)$$

$$\mu_{city}∼normal\left(0,1\right)$$

$$\alpha_{city},\alpha_{park},\alpha_{ind}∼e\text{xponential}\left(2\right)$$

The model was fitted using the No U-turn Sampler, an improved version of the Hamiltonian Monte Carlo algorithm in Stan (Gelman et al. 2015). A similar model was run for PC2.

## RESULTS

We traced a total of 3616 contact calls using Luscinia. This encompassed 1–4 days of recording effort and 2–756 recorded calls at each park, with a median of 48.5 calls (*n* = 28 parks). At the city level between 100 (Verona) and 701 calls (Brussels) were recorded, with a median of 459 calls (*n* = 8 cities). See Table 1 for additional sampling details.

**Figure 3:**
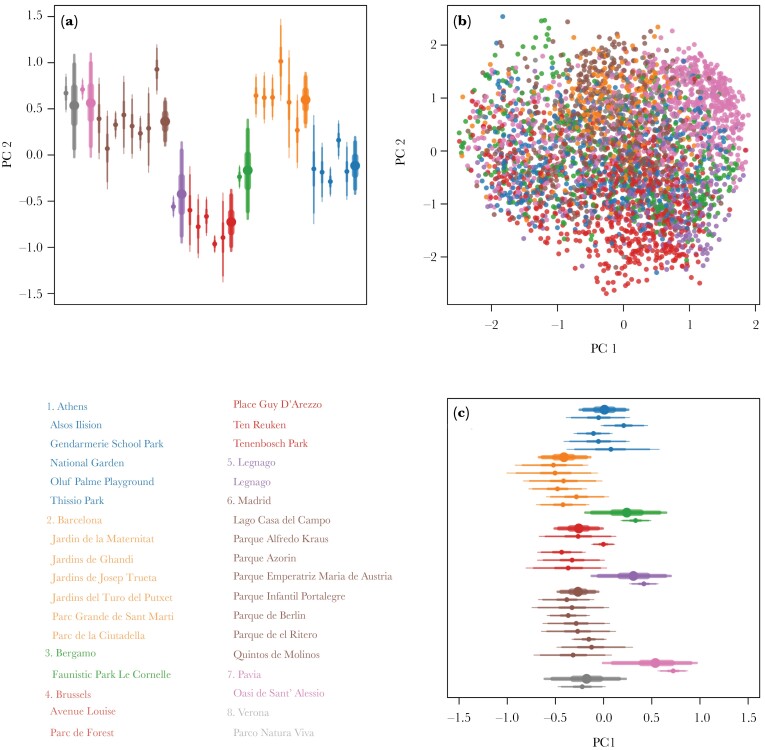
Result for PCO. Colors represent cities (see legend). a) City (thick) and park (thin) averages (dots) and 50, 90, and 95% intervals for PC 2. b) Scatter-plot of all calls included in the model. c) City (thick) and park (thin) averages (dots) and 50, 90 and 95% intervals for PC 1.

**Figure 4:**
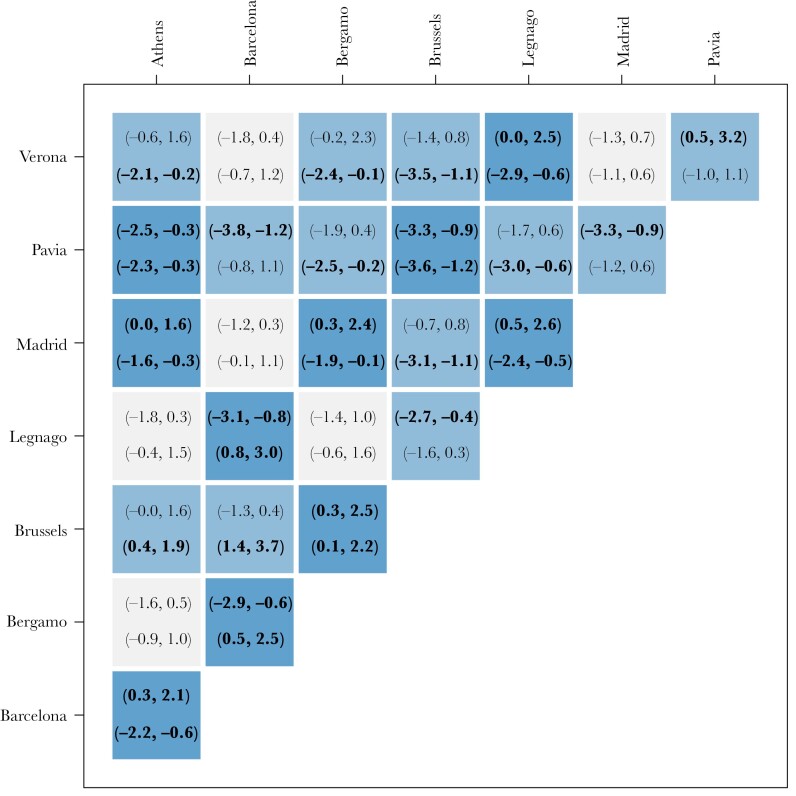
Pairwise contrasts between city means in principal coordinate space. Numbers in brackets give the 89% posterior interval for principal coordinate 1 (top) and 2 (bottom) for all city pairs. Intervals are in bold if they do not overlap 0. Squares are colored dark if either one (light blue) or both (dark blue) of the intervals do not overlap 0.

There was clustering by city (see Figure 3b) with distinct differences based on PC1 (see Figure 3c). In particular, Bergamo, Legnago and Pavia were different from the other cities (Figure 3c). For the second principal coordinate, the results demonstrated strong differentiation between the majority of cities (see Figure 3a). In general, there was considerable evidence that vocalizations differed between cities (mean σ_city_ PC 1: 0.40, 89% PI: 0.19–0.67, mean σ_city_ PC 2: 0.58, 89% PI: 0.34–0.92), and were less different between parks (mean σ_park_ PC 1: 0.21, 89% PI: 0.12–0.34, mean σ_park_ PC 2: 0.29, 89% PI: 0.19–0.42) as demonstrated by the sigma parameters and pair-wise contrasts (see Figure 4). These results were consistent across methods (see Supplementary Materials).

Differences between parks were only observed in a few cases. Lago Casa del Campo and Parque de el Ritero were clearly different from other parks in Madrid (see Figure 3a). Likewise, Gendarmerie School Park and the National Garden were different from other parks in Athens (see Figure 3a). It is important to mention that those observed park level differences could potentially be a result of incorrect pooling (i.e., assigning unique IDs to vocalizations from the same individual or assigning one ID to vocalizations from different individuals), as the standard deviation across parks was well within the values found in the sensitivity analysis (see Supplementary Figure S3). Park level means can appear very different under incorrect pooling, even when no signal exists in the simulated data (see Supplementary Figure S1). The city level signal we detected is much stronger than the simulated results due to incorrect pooling (see Supplementary Figure S2). This lends strong support for dialect differences between cities, while there is no support for this at the park level given the few differences observed.

**Figure 5:**
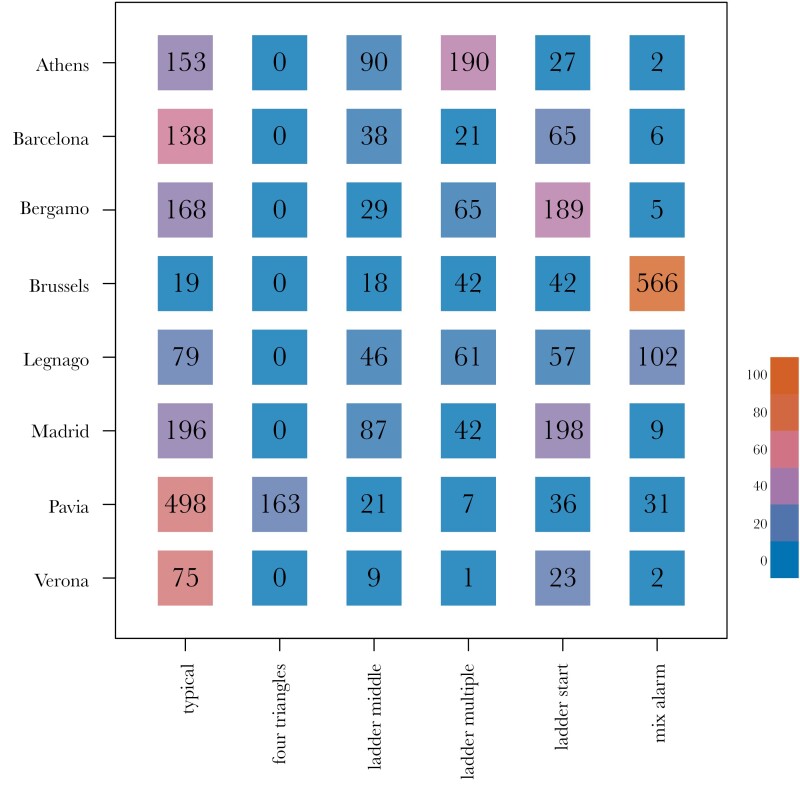
Distribution of variants across cities. Numbers are represented in black, colors represent the percentage of the given variant within the given city and range from 0% (blue) to 100% (orange)—see color scale bar.

In addition to assessing overall differences between parks and cities, we examined the proportion of contact call variants that were observed across the different cities (see Figure 5). We found that in most cities, the *typical* variant was prominent (see Figure 2a), and 4–5 other variants were usually present at intermediate to low frequencies. Multiple cities had a large proportion of contact calls that started with a low frequency component—*ladder start* (see Figure 2c). Pavia was characterized by a relatively high number of *four triangle* contact call with four triangular frequency modulations (see Figure 2b). Brussels stood out from the rest with the *mix alarm* contact call, containing multiple alarm-like notes (see Figure 2d).

## DISCUSSION

Our results provide strong evidence that monk parakeet contact calls differ between several cities that were sampled across their European range. Vocal differences between the parks within cities were also detected, however, these differences were less consistent compared to the dialect pattern we observed at the city level and appeared to be only present in a few parks (see Figure 3). Overall, our results provide support for the *cultural drift hypothesis*. While we do not explicitly find evidence against, we found no support for the *group membership hypothesis*. If vocal convergence was occurring at the group level, we would expect a stronger signal for dialects or clinal variation at the park level compared to city level, because movement between parks is likely very limited (Senar et al. 2021). Instead, our results demonstrate strong dialect differences at the city level. This result suggests that passive cultural processes are at play (Podos and Warren 2007; Bradbury and Balsby 2016; Sewall et al. 2016). Finally, while we cannot directly test this hypothesis in our framework, the lack of consistent evidence for park level differences is a pattern in line with other monk parakeet research (Smith-Vidaurre et al. 2020) that found strong support for the *individual signature hypothesis*. We should note that this is not mutually exclusive with the *cultural drift hypothesis*. It could be that both are operating simultaneously at different spatial scales (Thomsen et al. 2013), highlighting the importance of spatial scale in dialect studies.

Detecting the spatial scale at which geographic vocal variation emerges can be difficult, especially in a largely untagged population. For example, Smith-Vidaurre et al. (2020) used partial Mantel tests and detected a signal at all scales of their analysis. However, they were not able to directly compare this to the individual signal, as sample sizes differed and Mantel tests do not provide a comparable statistic. A Bayesian multilevel model does provide such a statistic (σ_park_ and σ_city_) and allows one to test the influence of incorrect pooling in a largely untagged population (see sensitivity analysis—Supplementary Materials). We can therefore say with a high degree of confidence that the city level signal outweighs the park level signal and is well above any spurious signal that might be due to incorrect pooling.

Previous studies in other parrot species have often argued that dialects arise at the group level because of selective pressures to conform to local variants (Wright and Dahlin 2018; Eberhard et al. 2022), including an active signaling of group membership. However, because we observed little evidence for dialects among parks, we do not think it likely that monk parakeets conform to local dialect types as a mechanism to identify group members. Instead, we find it more likely that the observed dialects among cities result from either random errors and conformity as described in the *cultural drift hypothesis*, or from an influence of the original founding populations (Ju et al. 2019).

This supports other work in parrots that has also found dialects all be it at smaller geographical scales (Wright 1996; Baker 2003; Kleeman and Gilardi 2005; Buhrman-Deever et al. 2007; Martínez and Logue 2020). Of course, we cannot exclude the possibility that low dispersal limits selection for local group dialects (Eberhard et al. 2022)

Given the limited dispersal between European populations of monk parrots, another possibility is that there is vocal and genetic concordance, as is observed in crimson rosellas (Ribot et al. 2012) and palm cockatoos (Keighley et al. 2020). However, we find this unlikely in our study system.

A previous study found that genetic differences between populations of monk parakeets in Europe are minimal, and that most areas were likely sourced from the same founding populations (Edelaar et al. 2015). Thus, genetic differences appear to be a less likely explanation for city level vocal differences than cultural processes, with the source groups determining the starting vocal dialect of each population. Even though previous work combined with our results suggest that monk parakeet contact calls are at least partially socially learned, the exact process is not fully understood and the ontogeny of vocal learning needs further study. It is well known that call structure of individuals is influenced by vertical transmission and the family environment (Berg et al. 2012; Berg et al. 2013; Arellano et al. 2022). Prior research suggests that dispersing juveniles are the ones most likely to modify their calls after dispersal while adults do not (Wright and Dorin 2001). However, we did not observe clear dialects at the park level, to which juveniles could converge.

Interestingly, previous research on invasive monk parakeets suggests that dispersal between both parks and cities is very limited (Dawson Pell et al. 2021). Hence, we might expect cultural drift to also lead to dialects at the park level, but we did not observe this pattern of differentiation.

Interestingly, we also found no support for clinal variation between parks (see further analysis in Supplementary Materials, where we tested the effect of distance on park-level vocal similarity). One possible explanation for why we do not observe dialects or geographic variation at the park level is provided by the *individual signature hypothesis*. Here, the lack of a clear park signature could be explained by divergence in order to stand out in acoustic space (Berg et al. 2011).

However, unlike the results from (Smith-Vidaurre et al. 2020), which suggest that selection for individually distinctive calls outweighs any selection for call convergence at the group level, we found very clear evidence for dialects between cities. A possible explanation for this discrepancy is that the study undertaken by Smith-Vidaurre et al. (2020) was undertaken in the native distribution of monk parakeets, while our results were obtained in a large invasive range where populations are fragmented and dispersal between populations (i.e., cities) is very unlikely (Borray-Escalante et al. 2023; Bucher et al. 1990; Dawson Pell et al. 2021). In contrast, although dispersal patterns have not been fully described in the native range, the habitat is more continuous, with increases in Eucalyptus trees allowing for long distance dispersal across the entire range (Da Silva et al. 2010; Bucher and Aramburú 2014). Furthermore, monk parakeets are considered an agricultural pest and are heavily persecuted in their native range (Castro et al. 2021). The effect of persecution is often increased dispersal and between-group movement (Payo-Payo et al. 2018) leading to increased intermixing between sub-populations that could potentially obscure any dialect patterns. Such differences in dispersal might partially explain why dialects were also detected in populations of invasive monk parakeets in the United States (Buhrman-Deever et al. 2007).

While we did not find evidence for strong convergence toward a group level signature in contact calls, it could be the case that group signatures exist in other call types, or within very specific variants of contact calls. In accordance to our call type analysis, (see Figure 5), most variants were present in all cities, but some showed higher proportions than others. While we cannot be certain that these variants drive the dialect differences between cities, or lack of in parks, they raise an important point. Explicit experiments that strive to determine the function of these can help us understand where and when to expect the stronger variation between them. Further complicating this, is that as vocal learners, it is possible that certain populations learn to use different variants in different contexts. The ontogeny of these variants, as well as the contextual mechanisms will help further the study of dialect mechanisms in not only monk parakeets, but all Psitticine species.

In Wright and Dorin (2001), it was found that juvenile birds more readily modified their contact calls after translocation than adult birds. Given that our populations started from invasive released birds, it could be a critical piece of information to know what the population dynamics were at the beginning of invasion, and the dynamics of subsequent invasion.

An alternative explanation for the lack of strong park signals could be that group signatures exist at a smaller scale. Monk parakeets nest in complex nest structures and previous work has shown that birds from the same nest tree are more closely related than expected by chance and tend to forage together (Dawson Pell et al. 2021). This might suggest that either passive or active processes could instead result in a nest level, rather than park level, signature. Future studies should focus on a single population and estimate the strength of the individual and group level signatures across multiple scales. This should preferentially be done in an individually marked population, such that the temporal stability of vocalizations can also be estimated. Lastly, we recommend that playback studies be conducted on monk parakeet across populations at both the park and city level to indeed experimentally test whether birds can detect subtle variations in group signatures, not picked up by our analyses. For example, tests could examine whether birds recognize calls from their own versus distant colonies, as well as other cities. Furthermore, playback tests could be used to test different substructures of the park (i.e., family unit, specific tree) to see if the park scale is an appropriate scale to measure vocal variation. This type of research is needed before dismissing the *group membership hypothesis* as a possible mechanism.

Geographic vocal variation is one of the primary forms of evidence for vocal learning (Marler and Tamura 1962; Lemon 1975). However, our understanding of the processes that lead to this variation at different scales and levels of population structure is lacking. A thorough understanding of these processes is critical to elucidating the underlying mechanisms that drive vocal learning and dialect formation. Monk parakeets and other parrot species are particularly useful model species to study social dynamics and vocal learning because of their flexible learning and complex social system. By continuing to apply novel techniques to the study of vocal patterns at different scales, we can uncover more detailed mechanisms of how communication systems evolve in natural populations. Our study demonstrates the existence of distinct dialects in European populations of monk parakeets, lending support to the *cultural drift hypothesis* while simultaneously showing patterns inconsistent with the *group membership hypothesis*. In addition to cultural drift, we also found evidence consistent with the *individual signature hypothesis* at the park level. While further experimental study is needed to confirm or refute these hypotheses, our extensive dataset, broad geographic scope and two-level comparison provide critical and robust information that enhances our understanding of the important role vocal learning plays in generating dialect differences among populations of Psittacine species.

## Supplementary Material

arad093_suppl_Supplemental_Results

arad093_suppl_Sensitivity_Analysis

## Data Availability

Analyses reported in this article can be reproduced using the code and data provided by Smeele et al. (2023a) and Smeele et al. (2023b).
